# 275 years of forestry meets genomics in *Pinus sylvestris*

**DOI:** 10.1111/eva.12809

**Published:** 2019-06-28

**Authors:** Tanja Pyhäjärvi, Sonja T Kujala, Outi Savolainen

**Affiliations:** ^1^ Department of Ecology and Genetics University of Oulu Oulu Finland; ^2^ Biocenter Oulu University of Oulu Oulu Finland; ^3^ Natural Resources Institute Finland Luke Oulu Finland

**Keywords:** adaptation, allele frequency spectrum, breeding, genomic prediction, genomics, inbreeding depression, linkage disequilibrium, *Pinus sylvestris*

## Abstract

*Pinus sylvestris* has a long history of basic and applied research that is relevant for both forestry and evolutionary studies. Its patterns of adaptive variation and role in forest economic and ecological systems have been studied extensively for nearly 275 years, detailed demography for a 100 years and mating system more than 50 years. However, its reference genome sequence is not yet available and genomic studies have been lagging compared to, for example, *Pinus taeda* and *Picea abies*, two other economically important conifers. Despite the lack of reference genome, many modern genomic methods are applicable for a more detailed look at its biological characteristics. For example, RNA‐seq has revealed a complex transcriptional landscape and targeted DNA sequencing displays an excess of rare variants and geographically homogenously distributed molecular genetic diversity. Current DNA and RNA resources can be used as a reference for gene expression studies, SNP discovery, and further targeted sequencing. In the future, specific consequences of the large genome size, such as functional effects of regulatory open chromatin regions and transposable elements, should be investigated more carefully. For forest breeding and long‐term management purposes, genomic data can help in assessing the genetic basis of inbreeding depression and the application of genomic tools for genomic prediction and relatedness estimates. Given the challenges of breeding (long generation time, no easy vegetative propagation) and the economic importance, application of genomic tools has a potential to have a considerable impact. Here, we explore how genomic characteristics of *P. sylvestris*, such as rare alleles and the low extent of linkage disequilibrium, impact the applicability and power of the tools.

## 1 INTRODUCTION

*Pinus sylvestris* (Scots pine) is one of the world's most widely distributed and northern conifers reaching from the British Isles in the west to the Siberian taiga in the east. It is also found in the mountainous areas of Mediterranean peninsulas in Southern Europe. It is especially competitive in poor soils and in dry and extremely cold environments, and compared to many other Pinus, its ecological niche is wide (Rehfeldt et al., 2002; Svenning, Normand, & Kageyama, 2008). Especially in the northern part of its distribution, it is a dominant tree in forested areas (Durrant, Rigo, & Caudullo, 2016) and has an important role as a part of forest ecosystems as a global carbon reservoir and also via interactions with soil microbes and fungi (Högberg et al., 2001; Lindén et al., 2014; Pan et al., 2011). Economically, it is an important tree species in Northern Eurasia as raw material for paper and pulp industry and as timber (Durrant et al., 2016; Gardner, 2013; Mason & Alía, 2000; Mullin et al., 2011). It is widely planted for timber outside its natural range. *P. sylvestris *is estimated to cover over 145 million hectares of forest in Eurasia (Durrant et al., 2016; Mason & Alía, 2000; Mullin et al., 2011).

Very rough estimates of the actual population census size can be made by combining distribution and density information. In commercial *P. sylvestris* forests, there are about 2000 stems per hectare after precommercial thinning (Fahlvik, Ekö, & Pettersson, 2005; Väisänen, Kellomäki, Oker‐Blom, & Valtonen, 1989), which yields an estimate of population census size of 290 × 10^9^ trees. *P. sylvestris* is not globally under threat in terms of species viability as such (Gardner, 2013). However, its dominant role in large forest ecosystems means that any changes in its distribution or mortality are likely to have large ecological and economic consequences.

Multiple subspecies and varieties of *P. sylvestris* have been described based on morphological and phenological differences (Molotkov & Patlaj, 1991; Pravdin, 1969; Ruby & Wright, 1976), but groupings are not consistent across studies and vary depending on the studied traits (Shutyaev & Giertych, 2000). Often the underlying phenotypic differences have more clinal rather than discrete distribution and the description of separate varieties reflects more the sampling design and silvicultural purposes than true biological groups (Langlet, 1971; Shutyaev & Giertych, 2000). At the genomewide level, molecular genetic differentiation among sampling locations at nuclear loci is low (*F*_ST_ = 0.02) in most parts of the distribution (Karhu et al., 1996; Kujala & Savolainen, 2012; Muona & Harju, 1989; Prus‐Głowacki, Urbaniak, Bujas, & Curtu, 2012; Pyhäjärvi et al., 2007; Tyrmi et al., 2019).

Due to its dominance and economical importance, phenotypic variation of *P. sylvestris* has been of interest since the early days of biological sciences. The first studies of phenotypic and adaptive differences among populations originating from different geographic regions were motivated by shipbuilding and forestry (Alberto et al., 2013; Engler, 1913; Langlet, 1971; Morgenstern, 1996). The first evidence of *P. sylvestris* provenance (common garden) trials in France comes from as early as 1745 (Langlet, 1971). Later, several variably documented national (Eiche, 1966; Heikinheimo, 1950) and international (Giertych & Oleksyn, 1992; Shutyaev & Giertych, 1998; Wiedemann, 1930) provenance trials have been established mainly for silvicultural purposes, also outside its natural range (Wright & Ira, 1962). Even though not originally designed for the purpose, these data have proved valuable for evaluating the level of ecological adaptation (Savolainen, Pyhäjärvi, & Knürr, 2007) and making predictions on responses to climate change (Persson, 1998; Rehfeldt et al., 2002). In the 20th century, very detailed studies on reproductive biology (Koski, 1970; Lönnroth, 1926; Sarvas, 1962) formed a solid basis for modern applied and evolutionary genetic research of *P. sylvestris*.

The extensive distribution, large effective population size (*N*_e_), efficient gene flow, predominantly outcrossing nature, and large 22 Gbp genome, yet strong phenotypic differentiation in adaptive traits, make *P. sylvestris* an intriguing system to examine adaptive processes of polygenic traits. Lack of significant longitudinal structure allows replicating sampling along latitudinal transects and inspecting whether the same loci or variants are participating in the adaptive trait variation throughout the distribution or whether the adaption has emerged via different combinations of adaptive alleles. In comparison with, for example, the North American interior spruce complex (Yeaman et al., 2016), *P. sylvestris* does not significantly hybridize with other species (see, however, Wachowiak & Prus‐Głowacki, 2008), likely because most close relatives have small distributions or do not overlap with *P. sylvestris*. Lack of hybridization should further facilitate understanding of the genetic basis of local adaptation. The species is also known to harbor large number of lethal equivalents (Kärkkäinen, Koski, & Savolainen, 1996; Koski, 1971), which allows examining the joint effects of deleterious variation with adaptive and phenotypic variations.

In comparison with farm animals, crops, and vegetables, many coniferous forest trees gene pools have not been extensively changed by humans, natural regeneration is common in large part of the distribution, and breeding is conducted in a handful of countries (Mullin et al., 2011). In Finland and Sweden, breeding efforts on *P. sylvestris* were started in the late 1940s by collecting phenotypically superior plus trees from natural and cultivated forests. Plus trees were used to establish seed orchards for seed production. Later, the genetic quality of the seed orchards has been improved by selection among plus trees based on progeny testing, forming the “1.5 generation” seed orchards (Haapanen, Hynynen, Ruotsalainen, Siipilehto, & Kilpeläinen, 2016; Jansson, Hansen, Haapanen, Kvaalen, & Steffenrem, 2017). With traditional breeding methods, genetic gains up to ~25% for growth traits like volume, and reduction in rotation times have been obtained and resulted in significant economic gains in Finland and Scandinavia (Ahtikoski, Ojansuu, Haapanen, Hynynen, & Kärkkäinen, 2012; Jansson et al., 2017).

Given the low number of breeding cycles conducted so far, there should be plenty of functional genetic variation, and thus, large genetic gains can be expected. By adding genomic methods to the available tools, we can expect further economic gains per unit of time. Genomic information can improve the breeding efforts in *P. sylvestris* through genomewide association analyses (GWAS), inference of genetic relatedness, and by genomic prediction (Isik, 2014; Isik et al., 2016; Meuwissen, Hayes, & Goddard, 2001). So far, efforts to make *P. sylvestris* to flower at earlier age have not been successful. Traits that can be measured only on adult trees hold greatest potential in terms of shortening the breeding cycle (Isik, 2014). Genomic prediction can deal with the highly polygenic basis of trait variation (with unknown underlying loci) unlike other breeding methods, such as gene editing, requiring detailed molecular information.

**Figure 1:**
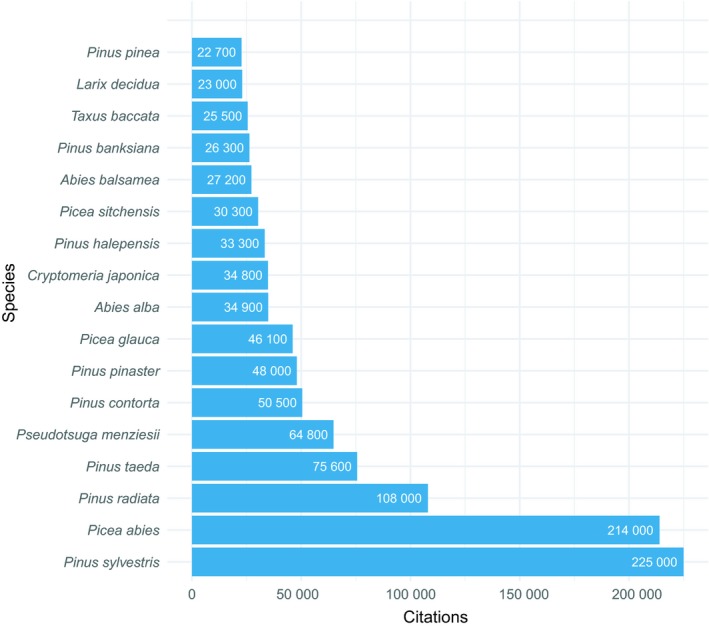
Number of approximate Google Scholar citations (as of October 19th 2018) for some most intensively studied conifer species. Number of results indicated in each bar

Given its economical (Praciak, 2013) and ecological importance, and the long record of research and knowledge (Giertych & Matyas, 1991), it is unfortunate that a whole genome sequence assembly of *P. sylvestris* has not yet been presented. Even though in many other ways, it is one of the most thoroughly studied conifers (Figure 1); from genomics point of view, it is still a nonmodel organism. However, whole genome sequencing and reference genomes are not a prerequisite for using current methodologies for genomic analysis of *P. sylvestris*.

In this review, we consider biological, life history, and genomic characteristics that are relevant for future applications of genomic information in *P. sylvestris* **.** We will address (a) mating system and inbreeding depression, (b) genetic diversity and linkage disequilibrium, (c) population structure, history, and allele frequency distribution, (d) adaptive variation at trait and molecular level, (e) genome size and architecture, and (f) application of genomics in breeding and conservation.

## 2 MATING SYSTEM AND INBREEDING DEPRESSION

*Pinus sylvestris*, as many other forest trees, is wind‐pollinated, diploid, and predominantly outcrossing. The selfing rate measured in mature seeds is about 5%–10% (Muona, Yazdani, & Rudin, 1987; Rudin, Muona, & Yazdani, 1986). *P. sylvestris* is also known to display high inbreeding depression, also a common observation in forest trees. Inbreeding depression is normally measured by comparing the performance of selfed (or other inbred) offspring to that of controlled outcrossed or open‐pollinated offspring. In pines, embryonic mortality can be evaluated by examining the proportion of empty seeds from different types of crosses (Kärkkäinen et al., 1996; Koski, 1971). The average mortality in selfed seeds is 75%–85% (Kärkkäinen et al., 1996; Koelewijn, Koski, & Savolainen, 1999; Koski, 1971), compared to 20%–30% for open‐pollinated seed. Based on these kinds of data, with simplifying assumptions, the number of embryonic lethal equivalents in *P. sylvestris* has been estimated to be 9.4 (Koski, 1971). The level is much higher than in most animals and angiosperms and similar to many other conifers, such as *Picea abies*, *Picea glauca,* or *Pseudotsuga menziesii*, while some pines with limited distributions have much lower levels (e.g., *Pinus radiata* and *Pinus resinosa*; Lynch & Walsh, 1998; Williams & Savolainen, 1996). The selfing rate at fertilization is thus much higher than at the mature seed stage. Further, the selfing rate at fertilization can be even higher because of polyzygotic (simple) polyembryony: An ovule may contain up to four embryos, which can be of selfed or outcrossed origin. These embryos share their maternal haplotype but have been fertilized by different pollen. Only one dominant embryo will survive (Hedrick & Muona, 1990; Koski, 1970; Sorensen, 1982). Inbred mortality during early embryogenesis and replacement by outcrossed progeny results in reduced loss of maternal resources, thus reducing post‐seed‐maturation inbreeding depression (Kärkkäinen & Savolainen, 1993; Sarvas, 1962).

Other studies (Koelewijn et al., 1999; Muona et al., 1987; Yazdani, Muona, Rudin, & Szmidt, 1985) have shown that the low fitness of selfs continue during later years, such that already in a few years old seedlings and especially in young adults, the selfed progeny has been mostly eliminated and the genotypes are in Hardy–Weinberg equilibrium. The results on conifers in general suggest that the overall inbreeding depression is due to a large number of deleterious, partially recessive alleles (Williams & Savolainen, 1996). Lande, Schemske, and Schultz (1994) showed that the lethals can be maintained in the population despite the selfing when there is a high per genome per generation mutation rate to the deleterious alleles. In addition, stabilizing selection on quantitative traits can interfere in complex ways with selection on lethals and either increase or decrease the probability of purging (Lande & Porcher, 2017).

Genomics can shed light on the genetic architecture of inbreeding depression by characterizing the numbers and effect sizes of deleterious alleles, for example, by mapping (Hedrick & Muona, 1990; Ritland, 1996), and by analyzing heterozygosity of the selfed progeny, as in *Eucalyptus grandis* (Hedrick, Hellsten, & Grattapaglia, 2016): In the selfed progeny, heterozygosity was much higher than the neutral expectation, suggesting that overall selection against homozygotes in selfed offspring was very high (*s* = 0.47). Furthermore, inbreeding depression likely was due to partially deleterious alleles at more than 100 loci, even if overdominance effects could not be fully excluded. Similar methods could help to identify genomic areas with lethals also in conifers. So far, various functional and evolutionary prediction models of allelic substitution have been used to identify deleterious alleles. This has then allowed comparisons of allelic and genotypic frequencies between populations or species, as in Populus (Zhang, Zhou, Bawa, Suren, & Holliday, 2016) or in Picea (Conte et al., 2017). Estimation of the distribution of fitness effects also allows conclusions on the nature of deleterious alleles. Allele frequency spectrum (AFS) of some 400 loci suggested that *P. sylvestris* has fewer slightly deleterious alleles and a larger proportion of more highly deleterious alleles than other conifers and plants in general (Grivet et al., 2017; Hodgins, Yeaman, Nurkowski, Rieseberg, & Aitken, 2016).

While mating between relatives can be an important breeding tool for some species, in conifers deleterious alleles are so numerous that breeding strategies using inbreeding will often lead to fixation of deleterious alleles, as shown in simulations by Wu, Hallingbäck, and Sánchez (2016). However, in some cases, variation in levels of inbreeding depression may allow using mating between relatives (Ford, McKeand, Jett, & Isik, 2014). Because of the mostly harmful effects of mating between relatives, it is important to manage inbreeding levels in breeding programs by measuring relatedness with genetic tools.

## 3 GENETIC DIVERSITY AND LINKAGE DISEQUILIBRIUM

In many aspects, patterns of genetic diversity in *P. sylvestris* match the expectations for a wind‐pollinated tree with large population size. Population structure is almost nonexistent, linkage disequilibrium (LD), the nonrandom association of alleles generally does not extend far, and genotypes are in Hardy–Weinberg equilibrium as expected under random mating (Kujala & Savolainen, 2012; Muona & Szmidt, 1985; Pyhäjärvi et al., 2007; Tyrmi et al., 2019; Wachowiak, Salmela, Ennos, Iason, & Cavers, 2011).

**Figure 2:**
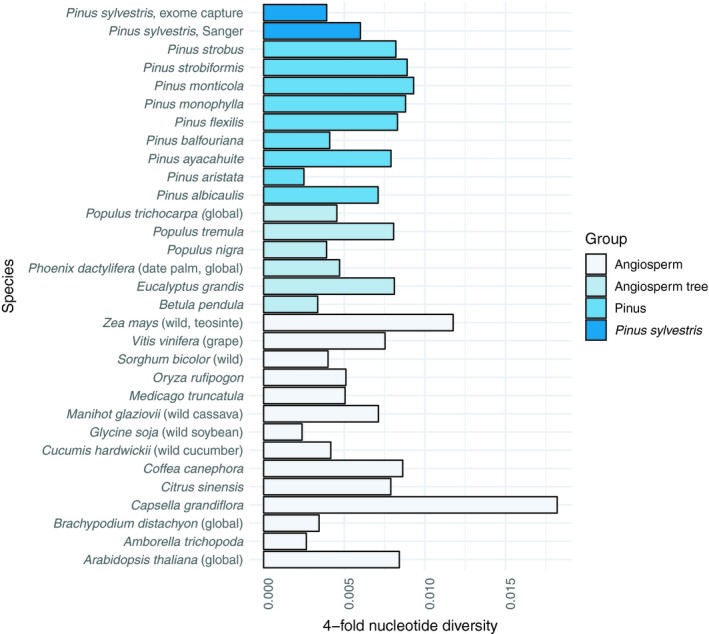
4‐fold site nucleotide diversity for variety of angiosperms and Pinus (data from Chen et al., 2017 except for *Pinus sylvestris*)

Comparative studies have indicated that *Pinus* in general do not have particularly low nucleotide diversity compared to other plants (Chen, Glémin, & Lascoux, 2017; Eckert et al., 2013; Figure 2). Silent site diversity in the *P. sylvestris* genic regions (based on Sanger sequencing) seems to converge to 0.006/bp (Dvornyk, Sirviö, Mikkonen, & Savolainen, 2002; García‐Gil, Mikkonen, & Savolainen, 2003; Grivet et al., 2017; Kujala & Savolainen, 2012; Pyhäjärvi et al., 2007; Wachowiak et al., 2011), and the first genomewide estimate of silent, fourfold degenerate sites genetic diversity is 0.004/bp (Tyrmi et al 2019). This estimate is slightly lower than the variation observed in other Pinus at fourfold degenerate sites (Eckert et al., 2013) and also slightly lower than diversity observed in silent sites of *P. sylvestris* in earlier studies. Note that even silent and fourfold degenerate nucleotide diversity and mutation rate estimates for *P. sylvestris* are based on data derived from genic regions. The overall patterns of diversity further away from genes may be very different and likely higher.

As for many other species, there is a mismatch between the observed nucleotide diversity and the census size in *P. sylvestris* (Lewontin, 1974). Under the assumption of standard neutral equilibrium, *4N_e_μ* (where *N_e_* is the effective population size and *μ* is mutation rate per site per generation) is expected to equal the pairwise nucleotide diversity, *θ* (Tajima, 1983). *N_e_* can then be estimated with *N_e_* = *θ*/(4*μ*). The mutation rates per bp per year vary from 0.22 to 1.3 × 10^−9^ (Buschiazzo, Ritland, Bohlmann, & Ritland, 2012; De La Torre, Li, Peer, & Ingvarsson, 2017; Pyhäjärvi et al., 2007; Willyard, Syring, Gernandt, Liston, & Cronn, 2007). Together with silent nucleotide diversity of 0.004/bp and assuming generation time of 20 years, this yields population size estimates ranging from 38,000 to 230,000 individuals. Yet, the actual census size is in the scale of billions of individuals in a seemingly random mating population. Clearly, some of the above‐mentioned assumptions are violated. Potential reasons for the discrepancy are as follows: (a) uneven fecundity among individual trees, which would increase the allele frequency variance across the generations, leading to increased drift, and deviations from the assumed Wright–Fisher population model; (b) much lower mutation rate than estimated based on fossil record and Picea–Pinus divergence; (c) nonequilibrium population history reducing the long‐term *N*_e_; and (d) effect of linked selection permeating most of the genic areas, thus reducing genetic diversity (Charlesworth, Morgan, & Charlesworth, 1993; Maynard Smith & Haigh, 1974).

These potential explanations do not exclude each other. It is likely that offspring number of *P. sylvestris* does not follow the assumed Poisson distribution with mean number of offspring = 1. Mature *P. sylvestris* trees have high fecundity, and thus, single trees have a potential to make a large contribution to next generation. This can lead to Moran model like multiple‐merger population coalescent processes, reduced diversity in relation to population census size, and also to an excess of rare alleles due to star‐shaped gene genealogies (Eldon & Wakeley, 2006). In seed orchard conditions, significant variation in offspring number has indeed been identified among genotypes (Gömöry, Bruchánik, & Longauer, 2003; Gömöry, Bruchanik, & Paule, 2000; Kang, Bila, Harju, & Lindgren, 2003; Savolainen, Kärkkäinen, Harju, Nikkanen, & Rusanen, 1993). In fully natural conditions, this variance is expected to be even larger. The AFS indicates nonequilibrium population history (Kujala & Savolainen, 2012; Pyhäjärvi et al., 2007), which also reduces the amount of genetic diversity. Linked selection has been shown to affect a wide spectrum of species (Charlesworth & Charlesworth, 2018), and evaluating its importance in *P. sylvestris* is one of the major evolutionary questions that more genomic data will help us to tackle. Grivet et al. (2017) observed both high efficiency of purifying selection and high rate of positive selection in *P. sylvestris* and *Pinus pinaster*, in accordance with gymnosperms in general (De La Torre et al., 2017), supporting linked selection as a likely explanation for low genetic diversity in *P. sylvestris* genic areas. Evaluation of the overall effect of linked selection on genetic diversity requires a dense genetic map combined with the physical map because a correlation between nucleotide diversity and recombination rate is expected. Current estimates suggest that LD in *P. sylvestris* in general decays fast, often within few hundred bp (however, see detailed discussion on LD below), which would predict local effects of linked selection nearby genic regions, as observed, for example, in maize that also has low LD (Beissinger et al., 2016). However, the partial selfing may generate some opportunity for linked selection, but this has not yet been studied.

Note that different genetic estimates and measures of nucleotide diversity may have apparent discrepancy due to scale. Some estimates such as nucleotide diversity are estimated at base‐pair resolution. However, other observations, such as lethal equivalents, reflect whole genome‐level phenomena. The genomewide mutation rate to deleterious alleles can be high even with low per base‐pair mutation rate level, if the mutational target size is large.

The recombination rate per bp (*c*) and extent of LD are critical factors in breeding as they determine how selection on a subset of loci will affect other nearby loci and essentially determines the genomic resolution of many breeding efforts. In an equilibrium situation, LD depends on both *c* and *N_e_* and the population‐level recombination parameter *ρ *= 4*N_e_c* be estimated from LD patterns. Further, *c* can be independently estimated with genetic mapping. In a practical context, it is good to remember that *c* affects the precision of, for example, QTL and other genetic mapping efforts, whereas *ρ* is more significant for association studies.

As mentioned above, *P. sylvestris* appears to have relatively low LD, when measured by *r* ^2^, squared correlation coefficient, extending the level above 0.2 often only just few hundreds of base pairs (Kujala & Savolainen, 2012; Pyhäjärvi et al., 2007; Tyrmi et al., 2019). The extent of LD measured with *r* ^2^ is useful for many applications, such as genomic prediction, because it directly informs about the power of a locus to predict the allelic state of another locus (Hahn, 2018). However, as all measures of LD, it is dependent on the marginal allele frequencies. When there are many low‐frequency SNPs, as in *P. sylvestris*, *r* ^2^ tends to be low not only due to high recombination but also due to low allele frequencies. *P. sylvestris* LD measured as |D′| (LD measure scaled by its maximum value given the allele frequencies) that is more informative about the recombination rate suggests more extensive LD than the value from the overall *r* ^2^ decay (Kujala & Savolainen, 2012).

As in most species, LD patterns in conifers are probably heterogeneous among different genomic regions (Pavy, Namroud, Gagnon, Isabel, & Bousquet, 2012). Despite the general trend of rapid LD decay (Pyhäjärvi et al., 2007; Wachowiak, Balk, & Savolainen, 2009), there is considerable variation along the genome in *P. sylvestris* at the gene level LD (Kujala & Savolainen, 2012; Pyhäjärvi, Kujala, & Savolainen, 2011). For example, Pyhäjärvi et al. (2011) found that several allozyme coding loci have strong LD, not showing signs of decay even over a 12‐kbp region. Also in *P. taeda*, earlier data suggested that LD decays rapidly (Brown, Gill, Kuntz, Langley, & Neale, 2004), but some recent work suggests large variation in the extent of LD in the genome (Lu et al., 2016, see, however, Acosta et al., 2019).

The overall recombination rate in conifers, estimated based on genetic maps, is in general low (Jaramillo‐Correa, Verdú, & González‐Martínez, 2010) and the same applies to *P. sylvestris*. The estimated map length is 1,500 cM (Komulainen et al., 2003) and with the genome size 22 × 10^9^ bp results in recombination rate of 0.07 cM/Mb or *c* = 0.7 × 10^−9^ per bp per generation. Thus, assuming *N*_e_ 38,000–230,000 obtained from nucleotide diversity data yields *ρ* estimates 0.0001–0.0006, whereas some *ρ*‐estimates obtained from the nucleotide diversity data are much higher ranging from 0 to 0.04 (Kujala & Savolainen, 2012; Pyhäjärvi et al., 2007, 2011; Wachowiak et al., 2009). The apparent discrepancy of low LD and low recombination rate can be explained by low minor allele frequencies, relatively high *N*_e_ and potential for low recombination and extensive LD in intergenic regions. Currently, LD estimates of *P. sylvestris* are available within genes and between pairs of coding areas. Evidence for LD in intergenic areas is rare, and studies at an intermediate range are missing in *P. sylvestris*. A study on *Cryptomeria japonica* has indeed shown that noncoding regions of conifer genomes can harbor extensive LD (Moritsuka et al., 2012). Better genome assemblies combined with extensive resequencing efforts are required to get a fuller picture of LD across *P. sylvestris* genome. Long read sequencing combined with, for example, optical and genetic mapping will allow identifying regions where physical and genetic distances have most discrepancies. Identifying these regions is important for breeding and understanding adaptive variation as large fragments of genome are dragged along when responding to selection.

## 4 POPULATION STRUCTURE, HISTORY, AND ALLELE FREQUENCY DISTRIBUTION

*Pinus sylvestris* has efficient wind pollination with potential for very long‐distance pollen dispersal. In addition, female flowering precedes the male flowering by two to five days (Koski, 1970; Varis, Pakkanen, Galofré, & Pulkkinen, 2009). *Pinus sylvestris *pollen dispersal distribution has a “fat‐tailed” leptokurtic shape allowing sporadic long‐distance dispersal events. Most pollen comes from nearby sources (Koski, 1970; Muona & Harju, 1989; Torimaru, Wang, Fries, Andersson, & Lindgren, 2009), but nonlocal airborne germinable pollen is often observed during the female flowering, and northern populations receive some southern pollen potentially from hundreds of kilometers away before the local pollen is available (Varis et al., 2009). In Robledo‐Arnuncio and Gil (2005), 4.3% of the pollen came outside the isolated *P. sylvestris* stand despite an estimated average pollen dispersal distance of only 135 m (Robledo‐Arnuncio & Gil, 2005). Even rare long‐distance dispersal can have an important role in homogenizing the distribution of genetic variation and result in, for example, suboptimal phenotypic variation. Seeds are also dispersed by wind, but not as extensively as pollen, distances peaking in <10 m (Kellomäki, Hänninen, Kolström, Kotisaari, & Pukkala, 1987).

The dispersal biology is reflected in the geographic distribution of genetic diversity. In nuclear genes that are dispersed by both pollen and seeds, the genetic differentiation is consistently low (*F*_ST_ = 0.02) in the more continuous part of the distribution (Karhu et al., 1996; Kujala & Savolainen, 2012; Prus‐Głowacki et al., 2012; Pyhäjärvi et al., 2007). Maternally inherited mitochondrial markers have a more restricted geographic distribution of alleles (*G*_ST_ = 0.66; Naydenov, Senneville, Beaulieu, Tremblay, & Bousquet, 2007; Pyhäjärvi, Salmela, & Savolainen, 2008). The eastern part of the distribution is less studied in terms of nuclear markers, but isozyme studies imply that genetic differentiation is also low (Dvornyk, 2001; Goncharenko, Silin, & Padutov, 1994). As in many other species, subtle geographic structure can be observed at the range margins and even in the main distribution when a large number of nuclear loci are observed (Kujala & Savolainen, 2012; Tyrmi et al.,^2019^; Wachowiak et al., 2011). In summary, the nuclear polymorphisms and continuous distribution indicate a lack of actual discrete populations, and in many analyses, *P. sylvestris* within most of Europe can be treated as a single panmictic population.

*Pinus sylvestris* distribution is continuous in the north, whereas in the southern margin, the distribution consists more of fragmented and isolated populations. Mediterranean peninsulas and Turkish populations harbor mitochondrial haplotypes that have rarely or never been observed outside these isolated populations (Cheddadi et al., 2006; Naydenov et al., 2007; Pyhäjärvi et al., 2008; Sinclair, Morman, & Ennos, 1999; Soranzo, Alia, Provan, & Powell, 2000; Wójkiewicz, Cavers, & Wachowiak, 2016). This reflects the existence of limited seed dispersal and Mediterranean refugia during the Last Glacial Maximum (LGM) and still ongoing postglacial changes in suitable habitats, but also contemporary land use and level of forested areas in general. In addition to contemporary gene flow, part of the low nuclear differentiation in higher latitudes could be explained by colonization process combined with a long juvenile stage (Austerlitz, Mariette, Machon, Gouyon, & Godelle, 2000).

**Figure 3:**
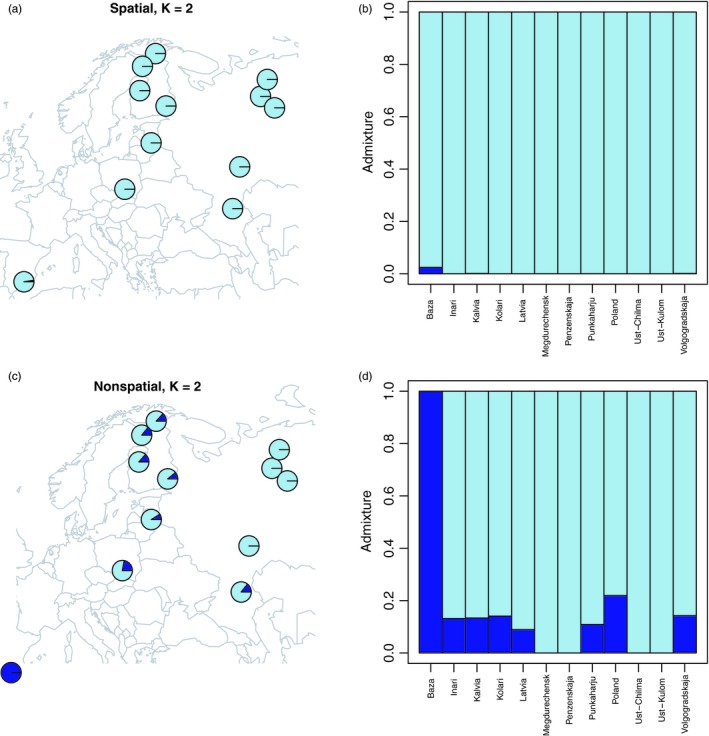
Application of spatial and nonspatial population structure model (Bradburd et al., 2018) with *K* = 2 to *Pinus sylvestris* exome capture data from (Tyrmi et al., 2019). Geographic distribution of layer contributions for each population under spatial (a) and nonspatial models (c). The proportion of layer contributions in each population for spatial (b) and nonspatial models (d). Sampling locations are ordered according to longitudes

Analysis of population genetic structure, measured as *F*_ST_ or inferred using STRUCTURE type approaches (Falush, Stephens, & Pritchard, 2003), is based on model of discrete populations. In reality, given the dispersal biology of *P. sylvestris*, the among‐population covariance structure is probably more of isolation‐by‐distance (IBD) type. Bradburd, Coop, and Ralph (2018) presented a promising new method to account for IBD patterns in genetic population structure analysis. When applied to nuclear *P. sylvestris* data, the spatial model with IBD has a better predictive accuracy than nonspatial (cluster) model (Tyrmi et al., 2019). The spatial model identifies a genetic component that is only present in an isolated Spanish population, consistent with previous work (Figure 3). However, in the spatial model the genetic makeup of the rest of the sampling sites remains continuous in contrast to the nonspatial model that assigns a proportion of western populations to the Spanish‐type cluster (Figure 3). These results support including IBD‐type spatial patterns in further analyses affected by population structure of *P. sylvestris* such as GWAS.

**Figure 4:**
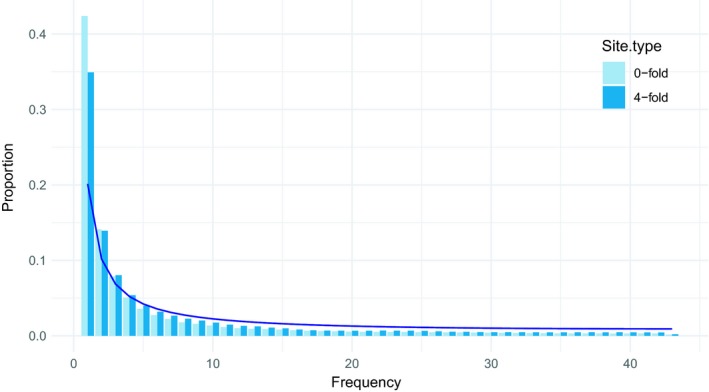
Example of an allele frequency spectrum in *Pinus sylvestris* (Tyrmi et al.,^2019^). Observed 4‐fold and 0‐fold degenerate sites are plotted as bars, and the expected values (Nordborg et al., 2005) under neutral equilibrium are indicated by line

Nucleotide diversity studies have revealed a nonequilibrium pattern in the distribution of allele frequencies of *P. sylvestris* throughout its distribution, which is a common observation in forest trees (Figure 4; Heuertz et al., 2006; Ingvarsson, 2008; Mosca et al., 2012; Zhou, Bawa, & Holliday, 2014). The observed excess of rare variants is consistent with a bottleneck whose timing is uncertain but much earlier than the LGM (Kujala & Savolainen, 2012; Pyhäjärvi et al., 2007).

**Figure 5:**
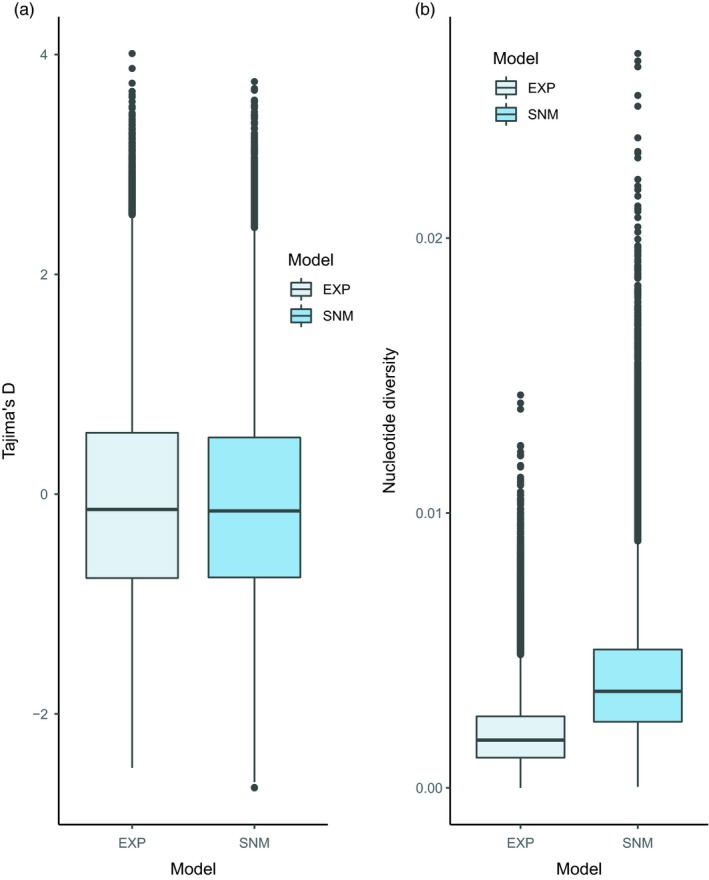
Tajima's D (a) and nucleotide diversity (b) distributions for constant size (SNM) and exponentially (EXP) growing population. Coalescent simulations were conducted with Coala package in R (Staab & Metzler, 2016) with the following parameters. SNM: *N*_0_ = 50 × 10^6^, *μ* = 0.004/bp and locus length 1,000 bp and sample size 50. EXP: as in SNM but exponential growth started at time 0.00002

As for many other conifers, the evolutionary timescales that influence both phenotypic and molecular genetic diversity in *P. sylvestris* can be very extensive, potentially reaching millions of years (Pyhäjärvi et al., 2007). This is a combined property of its long generation time and large *N*_e_. Therefore, observing the signatures of post‐LGM population expansion would require large amount of data, exceeding the sample sizes used in most published studies. Simple coalescent simulations show that, for example, doubling the population size from 25 × 10^6 ^to 50 × 10^6 ^individuals during the past 20,000 years (1,000 generations) hardly shifts the distribution of Tajima's D from the equilibrium population expectations (Figure 5). The large *N*_e_ results in long expected coalescence times, and thus, most of the observed diversity reflects the time before LGM. However, recent expansion could partly explain the observed low nucleotide diversity (Figure 5). It is noteworthy that during adaptation in rapidly growing populations, the current population size governs the adaptive process, whereas overall molecular diversity is defined by long‐term *N*_e_, which can be considerably lower (Messer & Petrov, 2013). Larger sample sizes, allowed by more affordable sequencing, will provide a better resolution on addressing more recent demographic events as, for example, in Keinan and Clark (2012).

The skewed AFS also has practical consequences. The general utility and information content of a given polymorphism depends on its allele frequency. Rare alleles are informative only in a small number of populations and families. Common alleles are generally considered more useful in, for example, paternity analysis, breeding, genetic mapping, genomic prediction, and genomewide association analysis. However, in some cases, ignoring the rare alleles may lead to biased conclusions. It will result in biased estimates of diversity and may lead to ignoring important functional variation (De La Torre et al., 2017, 2019; Fahrenkrog et al., 2017; Manolio et al., 2009).

## 5 ADAPTIVE VARIATION AT TRAIT AND MOLECULAR LEVEL

The extent of local adaptation and distribution of adaptive variation among geographic areas, genomes, and individuals is a core question in evolutionary genetics and also has impacts on conservation and breeding efforts. The ultimate proof of local adaptation is the superior fitness of a local population in comparison with nonlocal populations (Kawecki & Ebert, 2004). So far, the strongest evidence for local adaptation in *P. sylvestris* has been obtained at the phenotypic level, but modern tools contribute to connecting phenotypic with molecular variation. Many of the potentially adaptive traits display a continuous, nearly normal within‐population distribution indicative of polygenic basis of inheritance (Mather, 1943). Phenotypic variation is often also continuous at a geographic scale so that traits are correlated with, for example, altitude or latitude in a linear, clinal manner, caused by an interplay of selective gradient and gene flow in continuous space (Huxley, 1938). While many other conifer species have also been extensively characterized in terms of adaptive phenotypic variation, *P. sylvestris* is among the few for which extremely little population structure at genomewide level within a very large continuous distribution is coupled with high Q_ST_ values, measure of phenotypic differentiation among populations. These properties provide a relatively straightforward theoretical setup to study the dynamics of adaptive variation (Adrion, Hahn, & Cooper, 2015).

Barton (1999) suggests that a part of the loci affecting the clinal phenotypic variation should form strong allele frequency clines by the time population is approaching an equilibrium. Another suggestion emphasizes selection on favorable allele combinations across adaptive loci (allelic covariation) with only weak selection pressure on individual loci, instead of notable allele frequency clines. This could be especially important in the early phases of selection (Latta, 1998; Le Corre & Kremer, 2003, 2012). Further, phenotypes could well be genetically redundant, and allelic effects, transient (Barghi et al., 2019; Yeaman, 2015).

Over decades, *P. sylvestris* has been extensively characterized in terms of possible adaptive trait variation. In a comparison of 27 European conifer species, *P. sylvestris* had one of the highest *Q*_ST_ values on height increment, bud flush, and bud set (Alberto et al., 2013; see also Savolainen et al., 2007). Extensive phenotypic variation has been observed, for example, in phenology (Beuker, 1994; Clapham, Ekberg, Eriksson, Norell, & Vince‐Prue, 2002; Kujala et al., 2017; Mikola, 1982; Notivol, García‐Gil, Alia, & Savolainen, 2007; Salmela, Cavers, Cottrell, Iason, & Ennos, 2013), cold tolerance (Hurme, Repo, Savolainen, & Pääkkönen, 1997), drought (Palmroth et al., 1999; Semerci et al., 2017), waterlogging stress (Donnelly, Cavers, Cottrell, & Ennos, 2018), root (Zadworny, McCormack, Mucha, Reich, & Oleksyn, 2016), seed (Reich, Oleksyn, & Tjoelker, 1994) and needle (Donnelly, Cavers, Cottrell, & Ennos, 2016) characteristics, and carbohydrate and nutrient dynamics (Oleksyn, Reich, Zytkowiak, Karolewski, & Tjoelker, 2003; Oleksyn, Zytkowiak, Karolewski, Reich, & Tjoelker, 2000). For example, northern populations are more cold tolerant, have earlier growth cessation, and grow less during the growing season, consistent with the gradient in environmental conditions and local adaptation.

Wealth of data on survival and growth serving as proxies for fitness exists in provenance trials (reviewed by Langlet (1971)). Savolainen et al. (2007) used such data to identify local adaptation. Comparison of fitness of transferred populations to the fitness of local populations based on relative survival and height showed, for example, that *P. sylvestris* populations in central Sweden are locally adapted. This implies that tree populations have obviously had time to evolve to match the new habitats exposed by the retreating continental ice retreat (Davis & Shaw, 2001). Rapid adaptation is concordant with the theoretical expectation that selection is efficient in large populations.

The molecular genetic basis of the adaptive clinal variation can be identified with two basic approaches: association mapping that identifies genetic polymorphisms correlated with a given phenotypic variation, or by methods that rely solely on genotypic data. Association studies, especially when both the between‐population and within‐population variation can be addressed, can reveal important polymorphisms underlying adaptation. At the same time, the clinal setup poses a challenge for controlling population structure when it occurs along the same environmental gradient. Kujala et al. (2017) address this problem with latitudinal variation in timing of bud set in the first‐year seedlings of *P. sylvestris*. In this Bayesian approach, the presence of a within‐population association signal is required across different populations to exclude spurious associations. Other new promising methods, akin to *Q*_ST_−*F*_ST_ comparisons, for identification of polygenic adaptation rely on principal components of relatedness derived from genomic data and phenotype data obtained from common garden studies (Berg & Coop, 2014; Josephs, Berg, Ross‐Ibarra, & Coop, 2019, see however Berg et al., 2019). This type of analysis should also be now feasible in *P. sylvestris*, utilizing one of the available high‐throughput genotyping methods. The benefit of the Josephs et al. (2019) method is that it does not require a priori grouping of individuals into discrete populations and may be another solution to some of the analytical problems deriving from overcorrecting the population structure.

In *P. sylvestris*, association methods have been used to search for variants related to clinal variation in timing of bud set (Kujala et al., 2017). Most marker effects were small (<2 days), in line with most other genotype–phenotype association studies on growth cessation‐related traits in trees (Holliday, Ritland, & Aitken, 2010; Ma, Hall, St. Onge, Jansson, & Ingvarsson, 2010; Mahony et al., 2019; Olson et al., 2013; Prunier et al., 2013; Yeaman et al., 2016). Interestingly, in the *P. sylvestris* study, different markers were associated in northern and central European populations, suggesting genetic heterogeneity within this trait. While some sharing of the associated loci with other species has been found, the between‐species molecular convergence will be addressed in more detail in the upcoming association studies with higher genome coverage.

Divergence outlier methods (Beaumont & Nicholson, 1996; Excoffier, Hofer, & Foll, 2009; Foll & Gaggiotti, 2008) and deviations from the expected AFS have been used to identify loci indicating local adaptation or directional selection. However, inference of the effect of natural selection in *P. sylvestris* has been complicated by potential nonequilibrium demographic history. This has been accommodated by contrasting observed patterns of presumably neutral variation to a set of loci potentially under selection (Wachowiak et al., 2009). Another approach has been to build a demographic background model and derive expected patterns of neutral variation from that with coalescent simulations (Kujala & Savolainen, 2012; Pyhäjärvi et al., 2007). Even though most of these studies have been conducted on candidate genes, the number of outliers has usually been small (Kujala & Savolainen, 2012; Pyhäjärvi et al., 2007; Tyrmi et al., 2019; Wachowiak et al., 2009). However, despite moderate signals of selection at individual loci, Grivet et al. (2017) concluded that *P. sylvestris*, along with *P. pinaster*, have in general slightly higher substitution rates of adaptive mutations in comparison with many other plant species.

Genomes also carry evidence of very long‐term selection, for example, in d_N_/d_S_ ratios, as also identified in *P. sylvestris* (Palme, Pyhäjärvi, Wachowiak, & Savolainen, 2009; Palmé, Wright, & Savolainen, 2008). Identifying these kinds of “long‐term” selected loci is not necessarily helpful in current breeding efforts, but they will inform about the long‐term evolutionary forces shaping molecular patterns of, for example, divergence and transferability of the findings across species (Yeaman et al., 2016).

Very few nucleotide polymorphisms so far have indicated a clinal allele frequency pattern (Kujala & Savolainen, 2012; Tyrmi et al., 2019), and the overall lack of population‐level adaptive signal at molecular level is in strong contrast with the observed patterns of clinal phenotypic variation. This observation is consistent with the allelic covariation hypothesis, which would also explain the scarcity of other molecular level signal of adaptation. Alternatively, the clinal patterns could reside in yet‐unexplored genes or their regulatory regions. In the similarly adapted *P. abies*, some examples of latitudinal allele frequency variation have been found (Chen et al., 2012). A recent study on *Pinus contorta* shows that joint multivariate analysis of environment with gene co‐expression networks is a promising avenue to better grasp the architecture of polygenic clinal adaptation because in reality populations are adapting to different environmental dimensions at the same time, especially in complex landscapes (Lotterhos, Yeaman, Degner, Aitken, & Hodgins, 2018).

## 6 GENOME SIZE AND ARCHITECTURE

A major aspect of *P. sylvestris* genetics is its large genome size, 22 × 10^9^ bp (Bennett & Leitch, 2012). Assuming the composition is similar to the closely related *Pinus taeda* reference genome (Wegrzyn et al., 2014), only about 0.2% of its genome consists of coding regions. Pinus genomes have high repetitive content, extremely long introns (Stival Sena et al., 2014), large gene families, and potentially more than 50,000 genes (Stevens et al., 2016; Wegrzyn et al., 2014). A recent de novo transcriptome also indicates high complexity with more than 1.2 × 10^6^ distinct transcripts observed when multiple tissues and genotypes are combined (Ojeda Alayon et al., 2018).

The annotation of pine reference genomes is still far from complete (Wegrzyn et al, in the same issue). Current *P. taeda* (v. 2.01) and *P. lambertiana* annotations (Box ) reach at most 58% gene space completeness (Stevens et al., 2016) in terms of essential single‐copy genes in Embryophyta (Simão, Waterhouse, Ioannidis, Kriventseva, & Zdobnov, 2015). In contrast, more than 80% of the same essential genes are transcribed, for example, in the *P. sylvestris* tissues (Ojeda Alayon et al., 2018). The discrepancy is caused by still fragmented assemblies, extremely long introns, intronless genes, pseudogenes, and the inability of annotation algorithms to identify conifer genes (Neale et al., 2014; Nystedt et al., 2013; Stevens et al., 2016; Stival Sena et al., 2014; Wegrzyn et al., 2014). The incompleteness can cause biased results in downstream genetic analysis. For example, when read mapping of *P. sylvestris* is conducted using *P. taeda* as a reference, assuming polymorphisms in nonannotated sites to be intergenic or neutral may lead to biased estimates of genetic diversity or prevalence of natural selection.

Box 1 **Current genomic resources relevant for *Pinus sylvestris***

1 **Draft genome of *P. sylvestris* (** ftp://plantgenie.org/Data/ConGenIE/Picea_abies/v1.0/FASTA/GenomeAssemblies/Psylvestris_1Kbp.fa.gz, Nystedt et al. (2013)) contains 881,136 > 1 kbp unannotated contigs. 454 and Illumina raw data are downloadable in ENA database (project ERP002572).

De novo* P. sylvestris* **transcriptomes** are available for needles, pollen, vegetative buds, phloem, and various developmental stages of embryos and megagametophytes (Höllbacher, Schmitt, Hofer, Ferreira, & Lackner, 2017; Merino et al., 2016; Ojeda Alayon et al., 2018; Wachowiak, Trivedi, Perry, & Cavers, 2015). The haploid‐guided Trinity assembly combining data from multiple tissues and genotypes has the highest completeness (https://pinus_sylvestris_transcriptome_public_data.object.pouta.csc.fi/Pinus_sylvestris_transc riptomes_Ojeda_2018.tar).

**TreeGenes** (https://treegenesdb.org, Wegrzyn et al., 2008) provides access to several (including *P. taeda* and *P. lambertiana*) tree genomes, annotations, transcriptomes, and other resources and tools. It also provides *P. sylvestris* data obtained from NCBI for ESTs, cDNA, and TSA (Transcriptome Shotgun Assembly) databases. Somewhat overlapping with ConGenie.

**ConGenie** (http://congenie.org, Nystedt et al.) allows access to multiple conifer reference sequences, annotated genes, transcriptomes, and ESTs.

**PIER database** (Pine Interspersed Element Resource, https://www.treegenesdb.org/FTP/Genomes/Pita/Repeats/Pine_Interspersed_Repeats_%2528PIER%2529_v1.0.fa, Wegrzyn et al.) contains sequence information on 19,194 repeats first identified in *P. taeda*.

**Gymno PLAZA 1.0** (https://bioinformatics.psb.ugent.be/plaza/versions/gymno-plaza/, Proost et al., 2015) is a comparative genomics platform for 11 conifer species, including *P. sylvestris*. Information on gene families and orthological relationships among genes and species.

A set of **single‐copy genes** in gymnosperms, including *P. sylvestris* (http://bioinformatics.psb.ugent.be/supplementary_data/zheli/gbe/, Li et al., 2017).

**cpDNA genome and fragmented mtDNA genome sequences (** https://www.ebi.ac.uk/ena/data/view/PRJEB18435, Donnelly et al., 2016, https://www.ncbi.nlm.nih.gov/nuccore/JN854158.1, Parks et al., 2012 **)** can be used, for example, to identify sequencing reads originating from organellar genomes.

**STAPLER Pipeline maker** (Tyrmi, 2018) has been developed in conjunction with *P. sylvestris* exome capture workflow and functions with, for example, large *P. taeda* reference genome.

In addition to protein coding regions, conifers with large genomes may have relatively large functional space compared to, for example, angiosperm trees. There is likely a wealth of regulatory variation outside the coding region. Recent theory on the role of genome size suggests that large genomes have more potential for adaptive variation in noncoding region than small genomes (Mei, Stetter, Gates, Stitzer, & Ross‐Ibarra, 2018) and that the difference in mutational target can affect the expected dynamics of adaptation (Höllinger, Pennings, & Hermisson, 2019), which may partly explain why strong signals of adaptive variation in *P. sylvestris* genetic polymorphism (mostly targeting coding regions) have not been identified. Even though the genomic resources at the moment for *P. sylvestris* are limited, new techniques, such as ATAC‐seq, allow targeting the open chromatin and therefore access the regulatory regions of even large plant genomes (Buenrostro, Wu, Chang, & Greenleaf, 2015; Maher et al., 2018). The mutation rate per bp per year in Pinus is not extremely high (Buschiazzo et al., 2012), but the large functional space may also explain the large number of lethal equivalents (Koelewijn et al., 1999; Lande et al., 1994; Williams, 2009). The findings of both Avia, Kärkkäinen, Lagercrantz, and Savolainen (2014) and Alakärppä et al. (2018) on population‐specific expression patterns in *P. sylvestris* genes related to photoperiod circadian clock functions provide evidence of the importance of differential regulation. Interestingly, Alakärppä et al. (2018) also identified population‐specific expression patterns of DNA methyltransferase (DNMT) genes, encouraging further studies on expression regulations role in local adaptation.

Recently, the effects of transposable elements (TEs) on trait and adaptive variation via regulating gene expression have become evident across a wide variety of species (Chuong, Elde, & Feschotte, 2017). In large genomes filled with TEs, it is likely that adaptive mutations happen by TE movement and consequent epigenetic changes in regulatory regions (Mei et al., 2018). Transposable element sequences make up 80% of Pinus genomes in general (Stevens et al., 2016; Wegrzyn et al., 2014). Even if TEs are generally thought to be old and inactive, some are actively transcribed in certain tissues and conditions in Pinus (Cañas et al., 2017; Voronova, Belevich, Jansons, & Rungis, 2014). Similarly to other Pinus, *IFG‐7a_PTa* is the most abundant LTR family in *P. sylvestris* (Voronova, Belevich, Korica, & Rungis, 2017). Some families, such as *PtAngelina,* are especially abundant in *P. sylvestris* (Voronova et al., 2017), and several low copy retrotransposon family abundancies vary among *P. sylvestris* individuals. The intraspecific variation in TE abundancies indicates that they also have potential to relate to other intraspecific variation in, for example, adaptive traits or levels of inbreeding depression. Even though whole genome resequencing is still relatively expensive, a combination of low depth DNA sequencing and RNAseq can be used in more detailed studies on relative abundances of TEs and their activity across tissues, genotypes, and geographic regions.

One promising method for accessing genomic diversity in *P. sylvestris* is targeted sequencing where custom‐designed baits are used to target the regions of interest, such as exons in the genome. Baits can be designed by combining transcriptome data with information from reference genomes (Rellstab, Dauphin, Zoller, Brodbeck, & Gugerli, 2019; Suren et al., 2016). Tyrmi et al. 2019 have applied the method successfully in *P. sylvestris* and obtained good‐quality SNP data from 80,000 SNPs and 109 individuals with 30 Illumina HiSeq 2000 lanes. Even this approach has its challenges. With larger genomes, the off‐target sequence and paralog regions are harder to exclude (Rellstab et al., 2019). However, applying filtering for excess heterozygosity and deviations from expected read ratios have been successfully used to identify paralog mapping (different gene copies mapping to a single reference locus) in, for example, *Pinus cembra* (McKinney, Waples, Seeb, & Seeb, 2017; Rellstab et al., 2019).

As already done in reference genome sequencing and transcriptome sequencing (Ojeda Alayon et al., 2018; Stevens et al., 2016), it is advisable to utilize sequence data from the haploid megagametophyte tissue in the quality control, as no heterozygotes are expected, and paralog problems can be avoided. Heterozygous calls are not expected in haploid material, and they can be used to remove regions with paralog mapping issues. Utilizing haploid tissue also facilitates genotype calling with comparatively smaller average depth (10×) as there is no need to identify heterozygotes reliably.

RNA sequencing can also be used to identify polymorphisms (Ojeda Alayon et al., 2018; Wachowiak et al., 2015). However, one should proceed with caution in making detailed estimates of genetic diversity and AFS based on RNA‐seq due to, for example, allele‐specific expression. Ojeda Alayon et al. (2018) provide multiple *P. sylvestris* reference de novo transcriptome assemblies of which some are more suitable for use in gene expression studies and others for population genetic analysis, SNP identification, and, for example, exome capture bait design.

*Pinus taeda* can be used as a mapping reference for *P. sylvestris*, as the species have a rather low estimated sequence divergence of 3% at silent sites (Grivet et al., 2017; Palmé et al., 2008). Compared to *P. sylvestris* transcriptome and the draft genome sequence (Box ), it is probably the most comprehensive reference especially in the case when, for example, read data from targeted sequencing contain a lot of off‐target sequence not present in the transcriptome. Mapping to a diverged reference is bound to lead to some extent of reference bias and, for example, exclusion of highly diverged, potentially evolutionarily important genomic regions (Kronenberg et al., 2018 for an example in primates). This effect is likely to be even more serious in noncoding areas and further complicates identifying adaptive regulatory alleles that may be especially important in species with large genomes (Mei et al., 2018). *P. sylvestris*‐specific genome assembly would improve the quality of inference based on DNA sequence data and widen the available analytical repertoire (e.g., identification of copy number and structural variants, haplotype structure, and long‐distance LD). Box is a collection of useful genomic resources and databases available for *P. sylvestris* at the moment.

## 7 APPLICATION OF GENOMICS IN BREEDING AND CONSERVATION

Combining the phenotypic information from the phenotype and theory‐based knowledge with modern genomic technologies provides an opportunity for improved breeding methods and better predictions of *P. sylvestris* response to future environmental changes. Predicting responses to artificial or natural changes requires an understanding of both basic biological properties, evolutionary processes and genomic basis of adaptive trait variations, combined with practical aspects and reality of breeding and conservation activities. Two main methods, GWAS and genomic prediction, hold most potential to link genomic and phenotypic data together for application purposes.

GWAS uses large genomewide polymorphism and phenotypic data from typically large population samples to identify genomic variants associated with variation in a given trait and is based on tight LD between the marker and the causative locus (Risch & Merikangas, 1996; for trees, see Neale & Savolainen, 2004). So far, the association analyses in *P. sylvestris* have been based on a limited number of mostly candidate loci. Exome capture and transcriptome sequencing methods have now identified enough SNPs to enable design of efficient and affordable chip‐based genotyping methods (Stephen Cavers, personal communication). Also, genotyping‐by‐sequencing (Elshire et al., 2011) and other restriction‐based high‐throughput methods facilitate genotyping of hundreds of individuals for large amounts of SNPs. However, these methods have most power to detect SNPs in the genic, nonrepetitive part of the genome. If genetic basis of trait variation is highly polygenic and outside coding region, whole genome resequencing and targeted sequencing of active chromatin are the methods that offer better resolution. Both of these methods are still economically and technically challenging for conifers.

In a typical association study, the effects of rare variants are considered extremely hard to capture due to power issues. The lower the frequency of the variant is, the more samples are needed to reach sufficient power given a certain effect size. As the number of tests grows, the significance threshold grows due to corrections for multiple testing (however, note multilocus approaches, e.g., Kujala et al. (2017)). However, when the AFS is very skewed, and a minor allele frequency cutoff used, much genomic information is lost with rare variants, and the effects (small or large) of low‐frequency variants will not be evaluated at all (Auer & Lettre, 2015; Sazonovs & Barrett, 2018). The young variants are not efficiently tagged by older nearby variants due to the allele frequency difference (Auer & Lettre, 2015; Paulose, Hermisson, & Hallatschek, 2019; Sazonovs & Barrett, 2018; Wainschtein et al., 2019). Family‐based association methods, where the alleles that are rare in the general population are “amplified” within a given family (Laird & Lange, 2006), extreme phenotype sampling from the edges of the trait distribution (Guey et al., 2011; Li, Lewinger, Gauderman, Murcray, & Conti, 2011) or variance‐component tests (Lee, Abecasis, Boehnke, & Lin, 2014; Wu et al., 2011) can be used to increase power and efficiency in case of rare variants.

Genomic prediction is based on the idea that a limited number of genetic markers can inform about areas of the genome responsible for trait variation. It relies on extensive LD between genetic markers and the causal polymorphism, typically not observed at the population‐level samples. However, reduced *N*_e_ and family structure in breeding populations can increase the extent of LD. The observed LD patterns and empirical results in other conifers suggest that genomic prediction is feasible in cases where selection takes place within families (Chen et al., 2018; Lenz et al., 2017). *P. sylvestris* breeding, for example, in Finland is conducted in such a setting and allows genomic prediction to be developed.

The low success of transferring genomic prediction models across different breeding populations is due to differences in the patterns of LD between populations. This limitation has been observed in trees (Isik, 2014), as well as in other species (Meuwissen, Hayes, & Goddard, 2016). A dual strategy could alleviate this problem; instead of using an array consisting only of anonymous genotypic markers, one could allow more emphasis on markers in functionally relevant genes (Meuwissen et al., 2016; Nicolae et al., 2010; Wang et al., 2018; Zhang et al., 2015, 2010), for example, from well‐known pathways (such as pinosylvin biosynthesis; Lim et al., 2016). However, only those functional markers that have an impact on the variation across different populations would serve this purpose; markers with more locally distributed effects in contrary might enhance misinference. The above‐cited methods can however help to improve the genomic predictions within populations, too. Polymorphisms indicated in association studies in conifers are expected to be very close to the causal loci due to low LD, and these markers could thus be also given higher weight in genomic prediction. The excess of rare alleles is a challenge but also an opportunity for *P. sylvestris* breeding. Recent studies have shown that rare alleles may account for a considerable proportion of phenotypic variation and that some of these rare alleles may be deleterious, maintained by mutation‐selection balance (Wainschtein et al., 2019). There is definitely call for identifying and using rare variants also in plant breeding (Bernardo, 2016; Crossa et al., 2017). Further, they give highly accurate estimates of true genetic relationships and level of inbreeding (Eynard, Windig, Leroy, Van Binsbergen, & Calus, 2015). Jannink (2010) and Liu, Meuwissen, Sørensen, and Berg (2015) have shown that weighting low‐frequency favorable alleles increases the long‐term genetic gain in genomic prediction. The effect comes through reducing the odds of losing rare variation through genetic drift. This comes at the expense of short‐term genetic gain, but maintaining variation in the breeding populations is of high importance, too.

In *P. sylvestris*, each family or other subsample will have their unique set of rare variants. They are likely to be missed by, for example, general purpose SNP chips and can only be identified by sequencing‐based methods. Population stratification in rare alleles is likely to differ from the pattern observed with more common alleles as they often show more spatial clustering. Therefore, more rigorous methods to deal with the fine spatial structure are needed when working with rare variants (Mathieson & McVean, 2012). On the other hand, rare variants offer a way to learn more about the potential fine‐scale spatial structure in *P. sylvestris*, a topic not well covered so far.

Warming temperatures are already causing range shifts both at the northern and southern distribution limits of species, including *P. sylvestris* (Dyderski, Paź, Frelich, & Jagodziński, 2018). This is expected to increase growth rates of *P. sylvestris* in northern areas (Bärring, Berlin, & Andersson Gull, 2017; Bergh et al., 2003; Kellomäki et al., 2018). However, for this growth increase to be realized, forest reproductive materials with appropriate adaptive characteristics must be used (Beuker, 1994; Persson, 1998; Persson & Beuker, 1997; Rehfeldt et al., 2002) taking into account the complicated joint effects of abiotic and biotic changes (Matías & Jump, 2012). In some other areas, growth and survival may be reduced (Reich & Oleksyn, 2008). New combinations of photoperiod and temperature create selection pressure to efficiently utilize the increased potential for growth by correct interpretation of climatic signals (Saikkonen et al., 2012). When the genetics and relationship of phenotypic variation to environmental variation are understood, it is possible to predict and evaluate responses (Kuparinen, Savolainen, & Schurr, 2010; Savolainen, Bokma, García‐Gil, Komulainen, & Repo, 2004) and utilize this information, for example, in assisted migration design.

Along with the longer growth periods, also many forest disturbance effects are likely to take place. According to Seidl et al. (2017), temperature‐related disturbances will be highest in the boreal biome, especially in coniferous forests. Drought effects will be significant in both southern and northern regions, flooding will likely be an increasing problem, and new pests and pathogens will be encountered. It should be of high priority to assess the levels of within‐species and within‐population variation for adaptive potential against the predicted disturbances, at both phenotypic and genomic levels. Identification of the adaptive genomic regions, and diversity within those regions, albeit not an easy task, should be seen as an important task. Genetic analyses can also inform us, for example, about the extent of correlation among adaptively important traits, deriving either from pleiotropic effects or tight LD (Aitken, Yeaman, Holliday, Wang, & Curtis‐McLane, 2008; Savolainen et al., 2004). Further, genomic prediction may contribute to climate change responses helping meet goals of carbon neutrality (Cuny et al., 2015; Pan et al., 2011; Pool & Aquadro, 2007; Varho, Rautiainen, Peltonen, Niemi, & Ovaska, 2018) and alleviate associated disturbances (Alberto et al., 2013; Fady et al., 2016; Jansson et al., 2017; Savolainen et al., 2004; Seidl et al., 2017).

In summary, new genetic technologies and the molecular diversity they reveal—DNA polymorphisms, expression changes, structural, and repetitive element variation—are opening many venues to take a new look into questions identified by earlier *P. sylvestris* research and also applicable to many other forest trees. Bridging the artificial gap between quantitative, population, and evolutionary genetics is necessary for an in‐depth understanding of causes and consequences of, for example, clinal adaptive variation, inbreeding depression, large genome size, and skewed AFS in *P. sylvestris*.

## CONFLICT OF INTEREST

None declared.

## Data Availability

Data sharing is not applicable to this article as no new data were created or analyzed in this study.
